# Investigation into cases of hepatitis of unknown aetiology among young children, Scotland, 1 January 2022 to 12 April 2022

**DOI:** 10.2807/1560-7917.ES.2022.27.15.2200318

**Published:** 2022-04-14

**Authors:** Kimberly Marsh, Rachel Tayler, Louisa Pollock, Kirsty Roy, Fatim Lakha, Antonia Ho, David Henderson, Titus Divala, Sandra Currie, David Yirrell, Michael Lockhart, Maria K. Rossi, Nick Phin

**Affiliations:** 1 Public Health Scotland, Glasgow, United Kingdom; 2 Department of Paediatric Gastroenterology, Hepatology and Nutrition, Royal Hospital for Children, Glasgow, Glasgow, United Kingdom; 3 Department of Paediatric Infectious Diseases and Immunology, Royal Hospital for Children, Glasgow, United Kingdom; 4 Medical Research Council-University of Glasgow Centre for Virus Research, Glasgow, United Kingdom

**Keywords:** Hepatitis of unknown aetiology, outbreak, paediatric patients, adenovirus, public health response

## Abstract

On 31 March 2022, Public Health Scotland was alerted to five children aged 3–5 years admitted to hospital with severe hepatitis of unknown aetiology. Retrospective investigation identified eight additional cases aged 10 years and younger since 1 January 2022. Two pairs of cases have epidemiological links. Common viral hepatitis causes were excluded in those with available results. Five children were adenovirus PCR-positive. Other childhood viruses, including SARS-CoV-2, have been isolated. Investigations are ongoing, with new cases still presenting.

On 31 March 2022, the Scottish National Health Service (NHS) Greater Glasgow and Clyde (GGC) Health Board alerted Public Health Scotland (PHS) to five children aged 3–5 years presenting to the Royal Hospital for Children, Glasgow with severe hepatitis of unknown aetiology within a 3-week period. The typical number of cases of hepatitis of unknown aetiology across Scotland would be fewer than four per year [1].

This paper describes the initial investigation into the first Scottish cases and aims to raise awareness of this severe illness of unknown aetiology among young children.

## Early signal detection 

Following the alert, PHS convened a multi-disciplinary team of experts from across Scotland to review clinical and epidemiological data from the first five children presenting to hospital with acute severe hepatitis of unknown origin. Clinical case review revealed vomiting in preceding weeks, jaundice, and exceptionally high levels of alanine aminotransferase (ALT) among children of a similar age. Most children presented with transaminases greater than 2,000 international units per litre (IU/L) where the normal range is 10 to 40 IU/L [2]. Initial screening for hepatitis viruses A, B, C, and E was negative, with one hepatitis E result pending. One child had insufficient sample material to test for hepatitis B. As an indicator of disease severity, three children were transferred to quaternary paediatric liver units in England to be evaluated for liver transplant, with one receiving a transplant.

In addition to the clinical case review, the number of children presenting acutely with abnormal liver function tests in March 2022 to the Royal Hospital for Children, Glasgow were compared with those in March 2019 as well as March 2020 and 2021. These data confirmed higher-than-expected numbers in 2022 among children under 5 years of age, but not older children. As noted above, the number presenting in March 2022 at the Royal Hospital for Children also exceeded the total number expected for the whole of Scotland over 1 year [1]. 

On the basis of these reviews, PHS established a National Incident Management Team (IMT) to manage the ongoing public health investigation and response in Scotland. The National IMT developed a working case definition and active case finding was initiated, including a retrospective review of admissions to Scottish referral hospitals since 1 January 2022.

### Working case definition

A confirmed case includes anyone presenting since 1 January 2022 with aspartate transaminase (AST) or ALT greater than 500 IU/L of unknown cause who is either aged 10 years and under or who was a contact of any age of a possible or confirmed case. A possible case is defined as a person presenting since 1 January 2022 with jaundice without any known cause, either aged 10 years and under or who was a contact of any age of a possible or confirmed case. 

## Epidemiological investigations and outcomes

**Figure 1:**
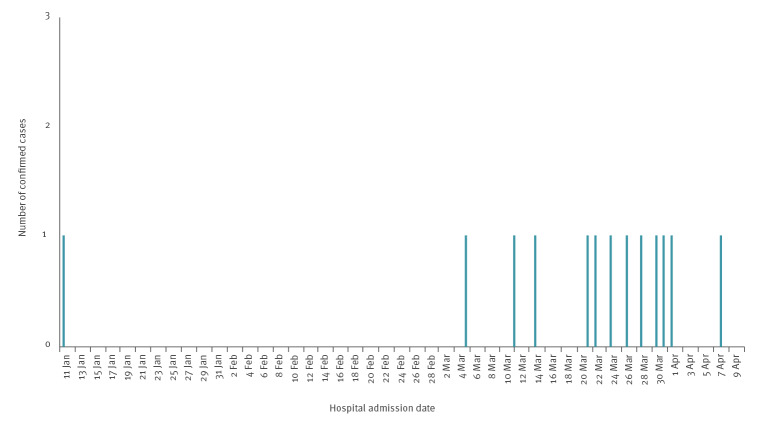
Epidemiological curve of date of hospital admission of confirmed cases of acute hepatitis of unknown origin in children 10 years of age and under, Scotland, 1 January–12 April 2022 (n = 13 cumulative cases)

As at 12 April 2022 (the submission date for this manuscript), 13 confirmed cases have been identified in Scotland, 12 of whom presented in March and April 2022 (Figure 1). No cases currently meet the possible case definition.

**Figure 2:**
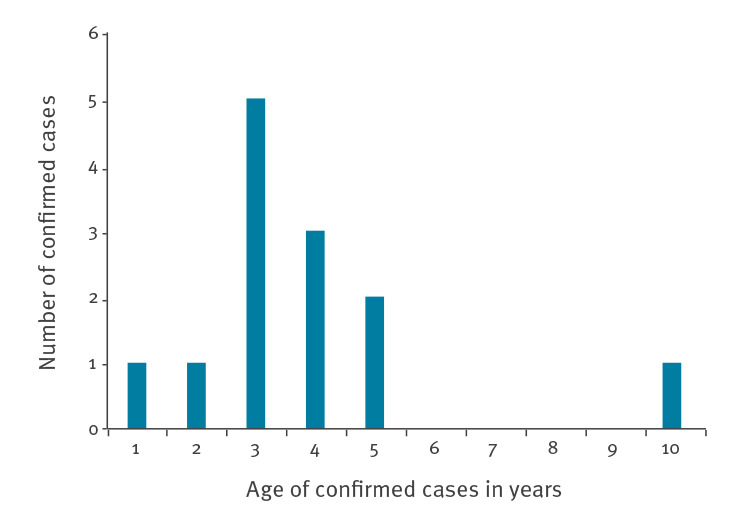
Confirmed cases of acute hepatitis of unknown origin in children, by age in years, Scotland, 1 January–12 April 2022 (n = 13 cumulative cases)

Median age of cases was 3.9 years (IQR: 3.6 to 4.6 years) (Figure 2). Seven of the 13 children were female; all were of white Scottish ethnicity. Children were primarily resident in central Scotland but with no otherwise discernible geographic pattern (e.g., living near a lake or river).

For nine of the 13 children parents/guardians completed the enhanced questionnaire to generate hypotheses about the aetiological source. For two children close contact in a household or other setting with two other cases was reported. Symptoms reported include jaundice (eight of nine cases), abdominal pain (seven of nine cases) and nausea and malaise (six of nine cases) leading up to admission. On 8 April, revisions to the enhanced questionnaire were made to ask about respiratory symptoms and pale stool. As a result, enquiries about these symptoms were made for four of the nine children with a submitted questionnaire. Nearly all of them reported gastrointestinal symptoms including diarrhoea or vomiting and lethargy, but not fever, in the weeks before admission.

As at publication, all cases had been admitted to hospital for a minimum of 6 days. Five still remain in hospital, including one in England who has successfully undergone a liver transplant but remains in hospital. Prior to their admission, cases were reported as generally healthy. None of them had any significant past medical history, such as an underlying immune deficiency, nor had they received immunosuppressive medication. One child had an underlying condition but was also previously reported to be in good health. 

## Laboratory investigations

All cases were tested for viruses commonly causing hepatitis among children (Table). Results available to date have been negative for hepatitis A, B, C and E viruses. Cases had bacteriology work ups, but as the majority did not have a fever, this line of investigation was not pursued unless clinically indicated by deranged blood inflammatory markers, including C-reactive protein or total white blood cell counts, or fever.

**Table:** Summary of viral tests conducted and results among children 10 years of age and under with acute hepatitis of unknown origin, Scotland, 1 January–12 April 2022 (n = 13)

Case identifier	Date of presentation 2022	Hepatitis A	Hepatitis B	Hepatitis C	Hepatitis E	SARS-CoV-2	Adenovirus	Other positive results
1	11 Jan	Negative (IgM) (31 Jan 2022)	Negative (12 Jan 2022)	Negative (Ab) (12 Jan 2022)	Not detected (12 Jan 2022)	Negative (12 Jan 2022)	Negative in blood (11 Jan 2022); Not tested by respiratory swab or stool	ASO < 20 (12 Jan 2022)
2	5 Mar	Negative (5 Mar 2022)	Insufficient material for initial test; subsequent positive (11 Apr 2022)	Negative (5 Mar 2022)	Negative PCR (8 Mar2022)	Negative (multiple tests taken 5–30 Mar 2022)	Initially negative in blood; Not tested by respiratory swab or stool; Tested positive (17 Mar 2022)	Equivocal for enterovirus (IgM) (8 Mar 2022); Low level positive for cytomegalovirus (urine PCR) (17 Mar 2022)
3	11 Mar	Negative (IgM) (14 Mar 2022)	Negative (14 Mar 2022)	Negative (Ab) (14 Mar 2022)	Negative (IgM) (25 Mar2022)	Negative (12 Mar 2022)	Pending	NA
4	14 Mar	Negative (IgM) (14 Mar 2022) (PCR) (15 Mar 2022)	Negative (14 Mar 2022)	Negative (14 Mar 2022)	Negative (IgM) (14 Mar 2022) (PCR) (15 Mar 2022)	Negative (three tests taken 14–22 Mar 2022)	Negative by stool (16 Mar 2022) and in blood (15 Mar 2022); Not tested by respiratory swab	NA
5	21 Mar	Negative (IgM) (22 Mar 2022) (PCR) (22 Mar 2022)	Negative (22 Mar 2022)	Negative (PCR) (22 Mar 2022)	Negative (IgM) (22 Mar 2022 and PCR 22 Mar 2022)	Historic positive (3 months before presentation on 29 Dec 2021)	Positive by respiratory swab (22 Mar 2022); Negative by stool (23 Mar 2022) and in blood (22 Mar 2022)	Human coronavirus NL63-positive (Ct value 34) (22 Mar 2022)
6	22 Mar	Negative (IgM and PCR) (22 Mar 2022)	Negative (23 Mar 2022)	Negative (PCR) (29 Mar 2022)	Negative (IgM) (22 Mar 2022 and PCR 26 Mar 2022)	Negative (five tests taken 22 Mar 2022─31 Mar 2022)	Negative by respiratory swab (31 Mar 2022) stool (23 Mar 2022) and in blood (23 Mar 2022)	NA
7	24 Mar	Negative (IgM) (4 Apr 2022)	Negative (4 Apr 2022)	Negative (PCR 4 Apr 2022)	Negative (IGM) (4 Apr 2022)	Positive (4 Apr 2022)	Negative by respiratory swab (4 Apr 2022) and in blood (4 Apr 2022); Stool not tested	Rhinovirus-positive (4 Apr 2022), Parainfluenza 2-positive (4 Apr 2022)
8	26 Mar	Negative (IgM) (27 Mar 2022)	Negative (27 Mar 2022)	Negative (Ab) (27 Mar 2022)	Negative (IgM) 27 Mar 2022)	Positive (point of care test on the day of admission, confirmed by PCR; 28 Mar 2022)	Positive by stool (28 Mar 2022); Negative by respiratory swab (26 Mar 2022) and in blood (30 Mar 2022)	Sapovirus-positive (23 Mar 2022); Parainfluenza 3-positive (2 Apr 2022)
9	28 Mar	Negative (IgM) (28 Mar 2022) (PCR) (29 Mar 2022)	Negative (28 Mar 2022)	Negative (PCR 2 Apr 2022)	Negative (IgM) (28 Mar 2022 and PCR 29 Mar 2022)	Positive (30 Mar 2022; point of care test not confirmed by PCR)	Positive by stool (30 Mar 2022); Negative by respiratory swab (2 Apr 2022) and in blood (29 Mar 2022)	NA
10	30 Mar	Negative (IgM) (PCR on 30 Mar 2022)	Negative (30 Mar 2022)	Negative (30 Mar 2022)	Negative (IgM) (PCR on 30 Mar 2022)	Historic positive (3 months before presentation 29 Dec 2021)	Negative by respiratory swab (1 Apr 2022), stool (2 Apr 2022) and in blood (30 Mar 2022)	NA
11	31 Mar	Negative (IgM) (7 Apr 2022)	Negative (7 Apr 2022)	Negative (7 Apr 2022)	Negative (IgM) (11 Apr 2022)	Negative (1 Apr 2022)	Positive in blood, negative by stool (1 Apr 2022); Respiratory swab not tested	Norovirus-positive (1 Apr 2022); Enterovirus/rhinovirus-positive (31 Mar 2022)
12	1 Apr	Negative (IgM) (2 Apr 2022)	Negative (2 Apr 2022)	Insufficient sample	Negative (IgM) (2 Apr 2022)	Negative (1 Apr 2022 and 2 Apr 2022)	Negative by respiratory swab, stool and in blood (2 Apr 2022)	Norovirus-positive (2 Apr 2022)
13	7 Apr	Negative (IgM) (10 Apr 2022	Negative (8 and 11 Apr 2022)	Negative (Ab) (8 and 11 Apr 2022)	Pending	Negative (8 Apr 2022 and 10 Apr 2022)	Pending	NA

Ab: antibody; ASO: Anti-streptolysin O; Ig: immunoglobulin; NA: no available data at the time of publication; SARS-CoV-2: severe acute respiratory syndrome coronavirus 2.

Patients admitted to hospital in Scotland are currently routinely tested for severe acute respiratory syndrome coronavirus 2 (SARS-CoV-2) [3]. Five of the 13 cases had a recent positive test—two that were 3 months before admission, two within 11 days of admission, and one point-of-care test was positive upon admission, which was not confirmed by PCR. Population prevalence of SARS-CoV-2 infection was highest in February and March 2022 at a time when the predominant variant in Scotland was the Omicron variant (Phylogenetic Assignment of Named Global Outbreak (Pango) lineage designation: B.1.1.529) sublineage BA.2 [4]. Modelled estimates of the percentage of the population testing positive for SARS-CoV-2 among those aged 2─4 years of age were around 7% in early February, peaking at around 13% in mid-March, and then declining to around 7% in early April [5].

**Figure 3:**
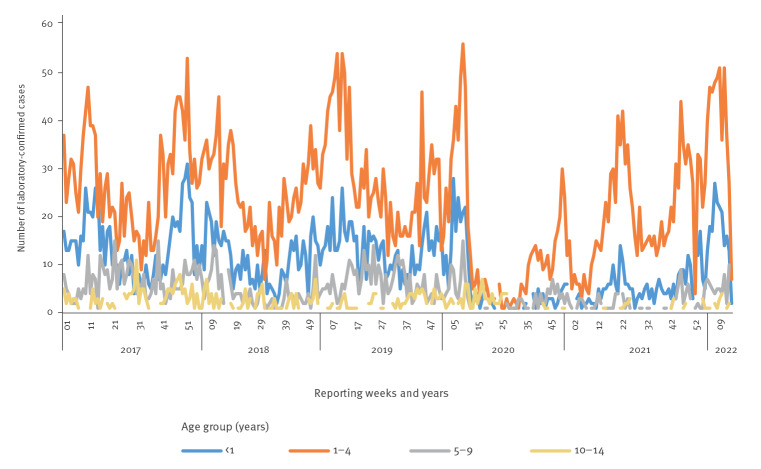
Laboratory-confirmed cases of adenovirus in children, by age groups, Scotland, 1 January 2017–6 April 2022

Additional viral testing not routine in hepatitis, but completed for some patients, included tests for enterovirus, parechovirus, human herpesvirus 6 and 7, varicella zoster and adenovirus. Five of 13 children were adenovirus-positive by PCR (two by throat swab, two by blood and one by stool). Adenovirus circulation in Scotland is seasonal, with peaks commonly seen between February and April (Figure 3). However, fewer cases were observed in 2020–21 because of physical distancing restrictions in place to mitigate SARS-CoV-2 transmission. Case numbers in March 2022 have returned to pre-pandemic peaks.

## Public health response

On 1 April 2022, PHS issued an alert to NHS Health Boards for awareness and described the actions to be undertaken for those meeting the case definition. On 6 April, PHS issued an alert to the public on the increased numbers of cases of hepatitis of unknown origin among children [6]. England, Wales and Northern Ireland published a similar communication [7]. As part of the wide public health response, PHS and the United Kingdom Health Security Agency (UKHSA) have been coordinating with the World Health Organization (WHO) and the European Centre for Disease Prevention and Control to alert other countries to this situation.

Consensus is being sought across the four nations in the UK on a standard set of laboratory tests to be undertaken should new cases present. In the interim, UKHSA has issued guidance on investigations that should be performed locally where available [7]. Additionally, PHS has collaborated with the International Severe Acute Respiratory and emerging Infection Consortium (ISARIC) Clinical Characterisation Protocol UK (CCP-UK) Study to recruit cases and identify and obtain residual and additional samples for further investigation of known and novel infections [8]. Non-invasive clinical samples also are being collected for any siblings resident in the same household if clinically unwell and select close contacts. Metagenomic sequencing and toxicology analyses are ongoing, however, results are not yet available. Investigation of milder disease in children as well as among adults is ongoing, although preliminary findings have not identified similar signals of severe outcomes or geographical clustering.

As warranted by the serious nature of the incident, UKHSA has established a Four Nations IMT and is coordinating and managing the ongoing investigation. Until the cause is identified, PHS has issued advice to strengthen standard infection control practices, which emphasise the importance of robust hand hygiene.

## Discussion

We report here on a cluster of cases of severe hepatitis of unknown origin in Scotland, mainly affecting children between the ages of 3–5 years. Approximately 60 cases have now been reported in England, Wales and Northern Ireland since 1 January 2022 [7]. PHS is also aware of a cluster of hepatitis and adenovirus cases among children being investigated by the US Centers for Disease Control and Prevention (personal communication: Hannah L. Kirking, 12 March 2022). 

In addition to the temporal clustering of these cases, the severity of disease upon presentation to hospital is unfortunately remarkable. At the time of publication, all 13 children had been hospitalised and three children required liver transplant evaluation in quaternary care centres in England, where specialist services are available. One child went on to receive a successful transplant. Five of the 13 cases are still being treated in hospital. To date, there have not been any fatalities. 

Initial hypotheses about the aetiological nature of the severe hepatitis included either an infectious pathogen or a toxic exposure to food, drinks or toys favoured by younger children. Toxicology analyses are ongoing among cases from across the UK, although responses to the enhanced investigation questionnaire about food, drink and personal care habits have not yet identified any common exposures. 

An infectious aetiology is now considered more likely given the epidemiological and clinical features, and taking into account the additional cases from across the UK and the US cluster. At the time of publication, the leading hypotheses centre around adenovirus—either a new variant with a distinct clinical syndrome or a routinely circulating variant that is more severely impacting younger children who are immunologically naïve. The latter scenario may be the result of restricted social mixing during the COVID-19 pandemic. Adenovirus infection as a cause of severe hepatitis is rare in immunocompetent children but has been reported in case reports and series [9-11].

Other infectious causes still being explored include increased severity of disease following infection with Omicron BA.2 (the dominant SARS-CoV-2 virus circulating in Scotland) or infection by an as yet uncharacterised SARS-CoV-2 variant. Of note, none of the children were vaccinated for SARS-CoV-2. A novel or yet undetected virus also cannot be ruled out at this time.

## Conclusion

We describe a clear and first signal in Scotland of the number of young children presenting to hospital with severe hepatitis of unknown origin. Rapid epidemiological analysis and laboratory testing is underway to identify the source. Public health specialists, paediatricians and other clinicians should be aware of children presenting acutely with gastrointestinal symptoms and jaundice or elevated serum transaminases > 500 UI/L (AST or ALT) and have a higher index of suspicion and a lower threshold for referral for specialist care. Cases should be reported to national and international public health bodies for appropriate follow-up investigation and management as well as the WHO for situational awareness. Until the cause of these cases of severe illness is found, standard public health guidance emphasising the importance of hand hygiene should be provided in settings where unexplained hepatitis is detected.
